# Edema as a Key Presentation of Acrodermatitis Chronica Atrophicans: A Retrospective Cohort Study from a Tertiary Setting in Denmark 2017–2025

**DOI:** 10.3390/diagnostics16091394

**Published:** 2026-05-05

**Authors:** Otto Scharff El-Nasser, Helene Mens, Nanna Skaarup Andersen, Christoffer Valdemar Nissen, Anne-Mette Lebech

**Affiliations:** 1 Department of Infectious Diseases, Copenhagen University Hospital-Rigshospitalet, 2100 Copenhagen, Denmark; otto.scharff.el-nasser@regionh.dk (O.S.E.-N.); anne-mette.lebech@regionh.dk (A.-M.L.); 2 Clinical Center for Emerging and Vector Borne Infections, Department of Clinical Microbiology, Odense University Hospital, 5000 Odense, Denmark; nanna.skaarup.andersen@rsyd.dk; 3 Research Unit of Clinical Microbiology, Faculty of Health Sciences, University of Southern Denmark, 5230 Odense, Denmark; 4 Department of Dermatology and Wound Healing Center, Copenhagen University Hospital-Bispebjerg and Frederiksberg, 2400 Copenhagen, Denmark; christoffer.valdemar.stoltenberg.nissen@regionh.dk; 5 Department of Clinical Medicine, Faculty of Health and Medical Sciences, University of Copenhagen, 1165 Copenhagen, Denmark

**Keywords:** acrodermatitis chronica atrophicans (ACA), borreliosis, lymphoedema, lyme, infection

## Abstract

**Background/Objectives:** Acrodermatitis chronica atrophicans (ACA), a late cutaneous manifestation of Lyme borreliosis, presents with a broad clinical spectrum. Most commonly, a characteristic bluish-red patchy rash, but it can also appear as unilateral limb swelling. This study aimed to characterize the clinical manifestations, diagnostic workup, and outcomes of patients with ACA in a tertiary setting in Denmark. **Methods:** Retrospective cohort study including all patients diagnosed with ACA at Copenhagen University Hospital-Rigshospitalet between 2017 and 2025. **Results:** Forty patients were included (median age 57 years; 63% female), with a median BMI of 24.5 [range 15.6–36.3]. Symptom duration was long (median 1 year). All patients presented with a skin rash. The most common location was the lower extremity, 26/40 (65%). Local edema and neuropathic pain were common (20/40) 50% and (23/40) 55%, respectively. A total of 13/40 patients underwent lymphoscintigraphy, which was deemed pathological in 7/13 (54%). The patients presenting with edema underwent significantly more imaging procedures, median 3 (range 1–5) vs. 0 (range 0–2), *p* < 0.005; they were younger, median age 49 years (range 17–76) vs. median 65 (range 30–81), *p* = 0.03; but did not differ in BMI, median 26.6 (range 19.0–36.2) versus median 23.8 (range 15.6–36.3), *p* = 0.48. All patients were *Borrelia burgdorferi* (Bb) IgG seropositive. *Borrelia*-specific PCR was positive in 6/13 (46%). Histopathology supported the diagnosis in 19/20 (95%). Clinical evaluation of the treatment response at 3 months was good in 33/40 (83%). **Conclusions:** Edema/swelling due to lymphatic obstruction is a common presentation of ACA in the tertiary setting, resulting in extensive diagnostic workup. The condition is associated with younger age but not BMI, sex, or immunodeficiency. Raised awareness and earlier testing for Bb IgG in serum seem warranted.

## 1. Introduction

Acrodermatitis Chronicum Atrophicans (ACA) is a late cutaneous manifestation of Lyme borreliosis (LB), a tick-borne infectious disease caused by spirochetes in the *Borrelia burgdorferi sensu lato* complex (Bb) [1]. Several decades prior to its identification as a tick-borne infection, the condition was described by Herxheimer and Hartmann in 1902 [2].

Lyme borreliosis is caused by several genospecies within the *Borrelia burgdorferi* sensu lato (s.l.) complex. Variations in clinical manifestations are strongly linked to specific genospecies, and for this reason, clinical presentations of Borrelia infection have geographical variations. In North America, *Borrelia burgdorferi* sensu stricto is the dominant genospecies. In contrast, *Borrelia afzelii* and *Borrelia garinii* are dominating in Europe. *Borrelia afzelii* is mostly associated with skin infections, whereas *B. garinii* is associated with neuroinfection [1]. Generally, but not exclusively, ACA is associated with infection by the genospecies *Borrelia afzelii* [3]. Although rare, cases of ACA have been reported from North America [4,5,6,7,8]. Although ACA arises from an initial skin infection, which for the most part is transferred in tick season, the diagnosis does not occur in a seasonal pattern due to the long incubation time, which can be months to years [1].

In Europe, the annual incidence of dermatologic manifestations ranges from 9.4 cases per 100,000 population in France to 155 cases per 100,000 population in Slovenia [9,10,11]. Acrodermatitis chronica atrophicans is estimated to constitute about 2.5–5% of all *Borrelia* manifestations [12,13].

The typical presentation of ACA is a slowly progressing red-bluish skin rash with or without skin atrophy or edema. Typically, the skin infection begins with an edematous-infiltrative stage (plasmacellular dermatitis), resulting in reddish discoloration of the skin, mostly unilaterally. With time, it transitions to the atrophic stage with purple to loam-brown discoloration of the skin, skin atrophy, loss of body hair, loss of connective and fatty tissues, protrusion of blood vessels, juxta-articular fibroid nodules, and joint involvement. It is associated with peripheral neuropathy in around 50% of cases, and a frequent complaint is pain and burning sensations in the affected area [14,15]. The location of ACA is most often on the extensor part of the distal extremities. ACA is associated with older age and female sex [16]. The diagnosis is based upon clinical recognition and supported by high levels of specific Bb IgG in serum [17]. A punch biopsy can further strengthen the diagnosis; typical findings of *Borrelia* infection are perivascular lymphocytic and/or plasma cell infiltrates. Spontaneous healing is exceptionally rare. Detection of Borrelia species by PCR and culture has limited sensitivity and is therefore not recommended for diagnosis [1].

However, skin changes may be subtle or non-specific, and swelling or pain is the main complaint. ACA may be mistaken for several other medical conditions such as chilblains, vascular insufficiency (chronic venous insufficiency), superficial thrombophlebitis, hypostatic eczema, arterial obliterative disease, acrocyanosis, livedo reticularis, lymphoedema, erythromelalgia, scleroderma lesions, rheumatoid nodules, gout (tophi), and erythema nodosum [1,18]. Furthermore, a tick bite or a preceding erythema chronicum migrans may not be recalled [19].

Once the diagnosis is established, the recommended treatment is a 21–28-day course of penicillin or doxycycline. However, no studies have evaluated the efficacy of antibiotic therapy duration or antibiotic type [20,21]. Despite effective antibiotic treatment, it is well documented that neuropathy and skin atrophy may persist [14,15,22]. Monitoring of treatment response may therefore be difficult; additionally, *Borrelia*-specific antibodies remain elevated for years [23]. The lack of progression may be the only hint of a favorable response [20].

The authors of this study work at a tertiary setting in Denmark. Over the years we have learned that ACA is a difficult clinical diagnosis, probably because of the variation in clinical presentation and rarity of the infection. We also noticed that a substantial part of patients in our setting presented with lymphedema, a clinical entity that is not well described in the literature of Borrelia skin manifestations. We therefore decided to characterize the spectrum of clinical presentation and treatment outcomes. Data were collected on clinical and paraclinical findings in patients diagnosed with ACA between 2017 and 2025 at the Department of Infectious Diseases, Rigshospitalet, Copenhagen. In this study we present the first systematic description of lymphoscintigraphic investigations in the context of ACA.

## 2. Materials and Methods

Study Design: A retrospective cohort study

Ethical Statement: According to Danish legislation, research projects using only register data do not require approval from the Regional Committees on Health Research Ethics (Region Hovedstaden). Informed consent from the patients was obtained to bring the Fotos.

Setting: The Unit of Tick-Borne Infections, Copenhagen University Hospital—Rigshospitalet, was established in 2017. Patients are referred from hospital departments and general practitioners for evaluation of tick-borne infections.

Study Participants: Patients diagnosed with ACA between 2017 and 2025 were included in the study. Inclusion criteria were a. being an adult (over 16 years of age) and b. being evaluated at the Unit of Tick-borne Infections, Copenhagen University Hospital, Rigshospitalet in the period 2017 to 2025 and c. being registered with a final diagnosis of ACA (registered as DA692A). An ACA diagnosis was based on a typical clinical presentation based on a combination of

- A chronic rash of violet, bluish-red, or reddish-brown color being either swollen, soft, and puffy or atrophic with a “cigarette paper” like appearance with acral location and
- Detection of Bb IgG antibody levels in serum.

In ambiguous cases, skin biopsies were taken for histological analysis and Bb DNA detection by PCR.

Patients were excluded if the diagnosis of ACA was obtained prior to the evaluation at the unit of tick-borne infections.

Rigshospitalet is a tertiary hospital setting where patients are referred from other hospital departments, dermatologists in private practice, and general practitioners.

Variables: The electronic patient records were assessed for information on basic characteristics such as age, sex, BMI, immunodeficiency, referral institution, location of skin lesions, unilateral or bilateral affection, edema, neuropathy, history of tick bite, antibiotic treatment, histopathological findings of skin, procedures as part of the diagnostic workup, including CT, MR, PET-CT, ultrasound with Doppler, standard ultrasound, lymphoscintigraphy, microbiological findings including the serum Bb IgG value, and finding of Bb-specific PCR of skin biopsies. Immunodeficiency was defined as receiving treatment with immunosuppressive chemotherapy or corticosteroids; solid or hematological cancer; alcohol abuse; diabetes mellitus; or congenital or acquired immunodeficiency, including human immunodeficiency virus infection.

Outcome: Outcome of treatment was based on clinical evaluation of the patient at the 3-months follow-up, categorized as good, moderate, or none based on the clinical assessment.

Data Sources/Measurements:

- *Borrelia*-Specific Antibodies: Serum Bb IgG was measured by the Liaison^®^ *Borrelia* IgG assay or the IDEATM assay and quantified by arbitrary units per milliliter (AU/mL).
- Histopathological Evaluation: A 3 mm punch skin biopsy was sent for pathological evaluation. The biopsy was deemed indicative of ACA if assessed as such by the describing pathologist.
- *Borrelia*-Specific PCR on Skin Biopsy: DNA was purified from a 3 mm skin biopsy using the MagNA Pure 96 system (Roche). The *Borrelia*-specific real-time PCR was carried out as previously described [24].

Bias: The study was carried out in a tertiary hospital setting; a higher proportion of atypical and severe clinical presentations is expected.

Study Size: All patients diagnosed with ACA at the unit of tick-borne infections at Rigshospitalet between 2017 and 2025 were included in the study.

Statistical Analyses: The R Core Team (2024, Vienna, Austria) software was used for descriptive statistics. Results are described in percentages, median, and range. A two-tailed Mann-Whitney U test was used for group comparisons.

## 3. Results

A total of 40 patients were diagnosed with ACA between 2017 and 2025. Baseline characteristics are shown in Table 1. The median age was 57 years, ranging from 17 to 81, with a slight overweight of females, 25/40 (63%). About half were referred from the Lymphedema Clinic, Bispebjerg Hospital, Copenhagen: 19/40 (48%), while others were referred from the department of infectious diseases, dermatologists in private practice, or general practitioners. The median duration of symptoms was 1 year, ranging from 0 to 14, and most had a history of a tick bite, 23/40 (58%). A minority were known to have immunocompromised conditions: 5/40 (13%). The dominating symptom was a skin rash occurring in 38/40 (95%), with edema and neuropathic pain reported in 20/40 (50%) and 23/40 (58%), while skin atrophy was rare in 4/40 (10%). The lower limbs were the most frequently affected body part in 26/40 (65%). A total of five patients were immunocompromised: this was due to anti-CD20 therapy (2/5), splenectomy (1/5), TNF-alpha inhibitor treatment (1/5), and well-treated HIV infection (1/5). None had severe immunodeficiency such as primary combined immunodeficiency, hematologic cancer, or organ transplantation.

Patients were mostly treated with doxycycline for 21 days, 35/40 (88%), and about a third received a second course of antibiotics within 3 months of the first antibiotic treatment, 12/40 (30%). The reasons for a second course of antibiotic treatment were as follows: a. no clinical response to antibiotic treatment after just 1 month’s evaluation (1/12, 8%), b. the patient had an insufficient treatment length of less than 21 days before the ACA diagnosis was settled (6/12, 50%); c. despite a good clinical response on ACA, the patient remained nervous a persistent borrelia infection (1/12, 8%); d. despite a good response to the ACA, other symptoms arose, and antibiotic treatment was given because the physician wanted to rule out a borrelia infection (3/12, 16%); e. persistent edema (1/12, 8%).

All patients were evaluated at the 3-month follow-up. Treatment outcome at 3 months following antibiotic treatment was evaluated as being good in 33/40 (83%). The five patients with compromised immunity all were evaluated as having a good outcome at the 3-month follow-up.

Microbiological and histopathological findings are shown in Table 2. A high Bb IgG level of >240 AU/mL was found in most patients, 22 of 40 (55%), whereas 9/40 had titers between 100 and 239 (23%), and 6/40 had titers under 100 (6%). In 2 cases, the titer was positive but not quantified. None had negative serology. A total of 13 patients underwent punch biopsy of the skin investigated by *Borrelia burgdorferi*-specific PCR. Interestingly, 6/13 were positive (46%). In only 1 of 6 was species identification carried out, confirming infection with *Borrelia afzelii*. A histopathological evaluation for signs of ACA was performed on 20 patients, with changes indicative of *Borrelia* infection in 19/20 (95%). The most frequent finding was perivascular inflammation with the presence of lymphocytes and plasma cells in the dermis.

The number and modalities of the diagnostic imaging workup are shown in Table 3. The most frequent diagnostic procedure was ultrasound with Doppler, carried out in 18/40 (48%), followed by ultrasound without Doppler 11/40 (28%), Tc-99m lymphoscintigraphy 13/40 (33%), CT thorax and abdomen 9/40 (23%), MRI 5/40 (13%), and PET-CT 4/40 (10%). Patients presenting with edema underwent a significantly higher number of diagnostic imaging procedures compared to those not presenting with edema, with a median of 3 imaging procedures (range 1–5) vs. a median of 0 (range 0–2), *p* < 0.05. Presenting with edema was not associated with immunodeficiency or sex. However, patients presenting with edema tended to be younger than patients presenting with rash (median 49 years (range 17–75) vs. median 65 years (range 30–81), *p* = 0.03 but did not differ in body mass index (BMI), median 26.6 (range 19.0–36.2) versus median 23.8 (range 15.6–36.3), *p* = 0.48.

**Figure 1:**
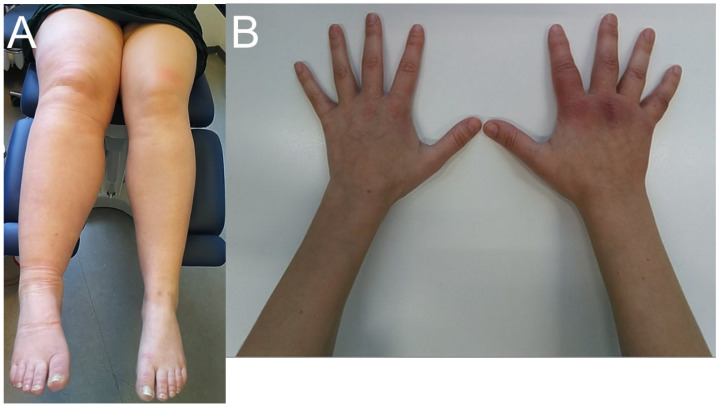
Different clinical presentations of Acrodermatitis chronica atrophicans. (**A**) a 41-year-old woman with dominating clinical presentation of edema in the right lower extremity, the rash is limited and almost not visible, (**B**) illustrates a 30-year-old woman with more classical ACA lesions located on the right hand with edema. Informed consent from the patients was obtained to take the Fotos.

Figure 1 illustrates differences in clinical presentations. On the left-hand side is a photo of a patient with lymphedema in the right leg, and on the right-hand side is a patient with a more classical presentation with a rash and edema located on the right hand.

Table 4 shows the results of the results of 13 patients who underwent technetium-99m lymphoscintigraphy. Interestingly, the lymphoscintigraphy was deemed pathological in 7/13 (54%), described with reduced transport capacity of less than 8% of the lymphatic vessels on the affected side in 3/7 (43%), and with signs of dermal backflow in 5/7 (71%).

## 4. Discussion

To our knowledge, this is the first retrospective cohort study on ACA from Denmark and the first study to date on ACA with detailed information on lymphoscintigraphic findings. About half of the patients presented with edema, undergoing a significantly higher number of diagnostic imaging procedures before the diagnosis was settled. Lymphoscintigraphy was described with dermal backflow in a handful of patients, suggesting that ACA not only involves reduced capacity but also structural incompetence of the lymphatic vessels. Our study underscores the importance of healthcare personnel being vigilant about *Borrelia* infection as a cause of lymphedema.

Edema in the context of ACA is well described. In the largest cohort of ACA cases to date, with extensive information on clinical presentation, containing 693 cases from Slovenia [16], a total of 194 of 693 (28%) reported swelling of the affected limb, most often the lower extremity. A Swedish study from 1998 reported 15 out of 111 (14%) presenting with swelling of the leg or foot causing shoe problems. A total of 49/111 patients diagnosed with ACA (44%) presented with bilateral affection, but it is not specified how many of these presented with swelling [25].

In our cohort, approximately half of the patients (20/40, 50%) presented with edema, a higher proportion than previously reported. In many of these cases the rash was atypical and not well defined. This likely reflects the tertiary hospital setting, in which atypical clinical presentations are to be expected. In our study, half of the patients were referred from the lymphedema clinic. While the overall disease pattern in Denmark is not expected to differ from other countries, these findings highlight the importance of considering ACA in patients with chronic edema. Primary lymphedema is usually diagnosed after excluding more common conditions such as cardiac insufficiency, chronic venous insufficiency, and cancer, which explains the extensive diagnostic imaging procedures performed. However, misdiagnosis may result in unnecessary and lifelong compression, despite the favorable treatment outcome for ACA [19]. Importantly, ACA should be considered in cases of a unilaterally swollen extremity once acute differential diagnoses are ruled out.

Lymphoscintigraphy is the gold standard when diagnosing lymphedema [26]. This dynamic investigation visualizes lymph flow and can identify malfunction of the lymph system. It is a hallmark of lymphedema if the lymphoscintigraphy shows fluid leakage to the superficial layers of the skin (dermal backflow). Moreover, a lymphoscintigraphy can quantify the transport capacity of the lymphatic system, which can also be a sign of lymphedema. Although it is established that ACA patients can exhibit edema, there are no other cohort studies describing lymphoscintigraphy in the context of ACA. The lymphoscintigraphic investigations in our cohort were interpreted as being pathological in half of the cases with asymmetrical and reduced transport capacity and/or tracer leaking back into the superficial dermis (dermal backflow), suggesting lymphatic obstruction or incompetence as a consequence of the borrelia infection.

It is well established that various infections can affect the lymphatic system and cause lymphedema. Most notable is the tropical disease lymphatic filariasis, which is the most common cause of secondary lymphedema in the world, affecting more than 51 million people [27]. In lymphatic filariasis, lymphedema is caused by parasitic nematodes (*Wuchereria bancrofti*, *Brugia malayi*, and *B. timori*) that can invade the lymphatic system and impair its function [28]. Erysipelas and cellulitis are other common causes of secondary lymphedema, especially in patients with recurrent disease. The mechanism for lymphedema in these patients is that each infection may lead to damage of cutaneous lymphatics, which can be visualized on a lymphoscintigram [29].

In *borrelia* infection, the development of lymphoedema is thought to reflect a convergence of pathological mechanisms driven by chronic infection of the dermis and subcutaneous tissues. Through activation of metalloproteases, the infection causes dermal fibrosis and atrophy [30]. The fibrosis compresses lymphatic capillaries. Additionally, the chronic Th2-skewed, profibrotic environment impairs lymphangiogenesis and augments the fibrotic process [8,31]. The chronic inflammation yields structurally abnormal and functionally insufficient lymphatic vessels that contribute to lymphatic stasis [32]. In some patients, fibrotic nodules—characterized by hyalinized collagen bundles and perivascular lymphoplasmacytic infiltrates—may further compound lymphatic obstruction through direct mechanical compression [18]. It is unknown why some patients with ACA present with lymphoedema as the dominating symptom. It has not been associated with risk factors. We found patients presenting with edema to be slightly younger. The condition was not associated with immunodeficiency.

A female predominance was found in this and previous studies on ACA [16,25], which is striking compared to Lyme neuroborreliosis, which has a clear male predominance [33]. Although it is beyond the scope of this study to elucidate sex-driven differences of the clinical presentations of Borrelia infection, one might speculate if they may be driven by women being more prone to developing a chronic Th2-skewed inflammatory response [34]. Currently, several anti-inflammatory drugs are being developed for type 2 inflammatory diseases, such as asthma and eczema, and it will be interesting if the addition of such therapies could optimize treatment response [35].

Although symptom duration was long, a median of 1 year and up to 14 years, antibiotic treatment outcomes were overwhelmingly positive, with the majority being evaluated as having a good response at the three-month follow-up. Our findings emphasize the significant benefit of diagnosis and treatment for ACA [22,36]. Although many reported a good outcome, the frequent need for more than one antibiotic treatment course likely reflects the slow resolution of ACA, rather than treatment failure. These findings emphasize the need for better and less invasive tools for diagnosis and monitoring of *Borrelia* infection. Proteome profiling has shown promising results in the setting of neuroborreliosis [37], potentially advancing diagnostics and response evaluation for ACA as well.

About half of the patients suffered from neuropathy, 20/40 (50%). This finding is consistent with the Slovenian cohort [16], where a total of 160/639 (23%) reported local symptom including pain, burning sensation, paresthesia, hypoesthesia, or itching. In the Swedish cohort, about 41% reported allodynia, noting that most patients (not quantified) experienced pain in the affected area [25]. Importantly, the characteristic pain reported by patients with ACA may be a hint to the diagnosis, especially if the rash is less pronounced.

Using the pan-borrelia PCR, about half of the investigated punch biopsies were positive. In only one case, sequence data was achieved, revealing infection with *B. Afzelii*. We found the PCR to be useful in establishing the diagnosis in some cases; however, given the low sensitivity, our study confirms that currently PCR cannot yet be relied on for diagnostics. Information on genospecies and the association to clinical disease is, however, still crucial for public health and better diagnostics, as most tick-borne infections do not show up in standard culture. In Europe, tick-borne infections have recently been increasing [38,39,40,41]. In Denmark we have observed an increase in cases of tick-borne encephalitis, Lyme neuroborreliosis, and tularemia as well as emerging pathogens *Neoehrlichia mikurensis* and *Borrelia miyamotoi* [42,43,44,45]. As tick-borne diseases are notoriously hard to diagnose, constant awareness of the changing epidemiological patterns is key to clinical recognition, and it is important that tick-borne infections or patients being evaluated for the presence of tick-borne infections are carried out at centers with a multidisciplinary approach and with clinical experience.

### Limitations

This study is limited by its observational nature, retrospective design, small sample size, and the fact that it was performed in a tertiary setting. Clinical outcomes were evaluated by unblinded clinicians, increasing the risk of bias. It should also be noted that the patient population of the one center may not be representative of the general population, which may limit the generalizability of the results. However, to the best of our knowledge, we present the first-ever data on the diagnostic workup for patients eventually diagnosed with ACA.

## 5. Conclusions

Edema/swelling is a common presentation of ACA in the tertiary setting, resulting in extensive diagnostic workup. Lymphatic obstruction or incompetence was evident by lymphoscintigraphy. Raised awareness of edema in ACA and earlier testing for Bb IgG in serum seems warranted, as well as improved diagnostics.

## Figures and Tables

**Table 1:** Baseline characteristics and treatment outcome (*N* = 40).

Median age (range)—yr	57 (17–81)
Sex at birth, female—no. (%)	25 (63)
Median BMI (range)	24.5 (15.6–36.3)
Median symptoms duration (range)—yr	1 (0–14)
History of tick bite—no. (%)	23 (58)
Immunocompromized *—no. (%)	5 (13)
Referred from the Lymphedema clinic—no. (%)	19 (48)
**ACA localization—no. (%)**	
Arm and leg	4 (10)
Isolated arm	8 (20)
Isolated leg	26 (65)
Bilateral involvement (leg)	3 (8)
Bilateral involvement (arm)	1 (3)
Other location than extremity	5 (13)
**Clinical presentation—no. (%)**	
Neuropathic pain	23 (58)
Edema	20 (50)
Skin rash	38 (95)
Atrophy	4 (10)
**Antibiotic treatment—no. (%)**	
Doxycycline 21 days	35 (88)
Penicillin 21 days	5 (13)
Prolonged antibiotics †	12 (30)
**Treatment outcome—no. (%)**	
Good	33 (83)
Moderate	3 (8)
None	4 (10)

Abbreviations: BMI: body mass index, yr: years, no.: numbers, %: percentage, ACA: acrodermatitis chronicum atrophicans, * Immunodeficiency includes immunosuppressive chemotherapy, biologic treatment (including B-cell depleting therapy and TNF-Alpha inhibitors), corticosteroids, solid or hematological cancer, organ transplant recipients, diabetes, asplenia, primary immunodeficiency, and HIV infection. † More than 1 course of 3 weeks of anti-borrelial antibiotics within 3 months of presentation.

**Table 2:** *Borrelia* diagnostics and histopathological findings among patients with ACA.

Borrelia Titer AU/mL—no. (%) (*N* = 40)	
>240	23 (58)
100–239	9 (23)
<100	6 (15)
Positive, not quantified	2 (5)
PCR on skin—no. (%) (*N* = 13)	
Positive	6 (46)
Negative	7 (54)
Histology (*N* = 20)	
Indicative of ACA—no. (%)	19 (95)

Abbreviations: no.: number, %: percentage, AU: arbitrary unit, mL: milliliter, >: greater than, < less than, PCR: polymerase chain reaction, ACA: acrodermatitis chronicum atrophicans.

**Table 3:** Diagnostic imaging workup among patients with ACA (*N* = 40).

Diagnostic Imaging	No. (%)	*p*-Value *
Ultrasound with Doppler	18 (45)	
Ultrasound without Doppler	11 (28)	
Tc-99m lymphoscintigraphy	13 (33)	
CT thorax/abdomen	9 (23)	
MRI extremity	5 (13)	
PET-CT	4 (10)	
**Median no. diagnostic imaging procedures per patient (range)**		
Presenting with edema (*N* = 20)	3 (1–5)	<0.05
Presenting with rash (*N* = 20)	0 (0–2)	

Abbreviations: Tc-99m: Technetium-99m; PET-CT: Positron emission tomography-computed tomography; No.: number; %: percentage; MRI: magnetic resonance imaging. * Mann-Whitney U test.

**Table 4:** Results of Tc-99m lymphoscintigraphy (*N* = 13).

Age (yrs.)	Sex	Location of ACA	Lymphedema *	Transport Capacity † of Affected Limb	Dermal Backflow
20	F	Right leg	Yes	NA	Yes
41	F	Right leg	Yes	4.4%	Yes
34	M	Right leg	Yes	35%	Yes
62	F	Right leg	Yes	1%	Yes
64	F	Left leg	Yes	6%	No
48	M	Left leg	Yes	NA	Yes
59	M	Left leg	Yes	NA	No
49	F	Left leg	No	13.9%	No
26	F	Right arm	No	NA	No
40	M	Both arms	No	NA	No
63	F	Left leg	No	20.7%	No
49	F	Both legs	No	24.1%	No
67	M	Left leg	No	5%	No

Abbreviations: Tc-99m: Technetium-99m; F: female; M: male; NA: not available—either due to technical difficulties or performed at another hospital. * The overall conclusion was lymphedema, according to the describing nuclear medicine physician. † Estimated 2 h after injection of the tracer, values above 8% are considered normal.

## Data Availability

The data are not publicly available due to legal reasons. Data will be available from the authors upon request.

## Abbreviations

The following abbreviations are used in this manuscript:

ACA	Acrodermatitis Chronica Atrophicans
Bb	*Borrelia burgdorferi*
